# Applications of Celluloid

**Published:** 1879-07

**Authors:** 


### ARTICLE VI.

### *Applications of Celluloid.*

An excellent illustration of the industrial and commercial benefits that may rise from new products, whether gleaned from the unused stores of nature or created by the skill of the inventor, is furnished by the wide and various utility of the compound of cellulose and camphor known as celluloid. Though scarcely ten years have passed since the Hyatt brothers suspected that this compound might be used profitably in the arts, and only five years since they began to manufacture it successfully, it has become the basis of several thriving industries, and novel applications of it are being made almost daily.

As now made celluloid is a composition of fine tissue paper and gum camphor, treated with chemicals by a patented process. When crude it looks like a transparent gum, and its color is a light yellow brown. It can be made as hard as ivory, but is always elastic, and can be readily moulded into every conceivable form. With equal ease it can be colored in any tint desired, the dye running through the entire substance, and being, therefore, ineffaceable.

A writer for the *Evening Post* has taken pains to collect a large amount of information concerning the manufacture and use of this material; and wide as the range of its application has become, the business of preparing the crude material and shaping it into novel and useful forms is thought to be only in its infancy. According to the *Post* writer, all the celluloid used is made by a single company, having factories at Newark, N. J., who sell the crude material to the parties undertaking the production of finished goods. No one can buy it unless the producing company decides to give him a license, which is granted only for the purpose of making some new article that will not interfere with the trade of the companies already licensed. A number of large corporations are now engaged in the various branches of manufacture for which celluloid can be employed. Most of these have their factories in Newark, but there is one large establishment in Center street, this city.

The cost of the crude article to the buyers is regulated by the producing company according to the use to be made of it and the competition met with in other materials. For instance, $4 or $5 per pound are charged for celluloid which is to be made into jewelry, while only $2 are charged if it is designed for umbrella handles, though there is no difference in the quality of the substance.

As a close imitation of ivory, celluloid has made great inroads in the business of the ivory manufacturers. Its makers assert that in durability it is much superior to ivory, as it sustains hard knocks without injury, and is not discolored by age or use. Great quantities of it are used for piano and organ keys, to the manufacture of which one company is devoted.

Billiard balls are made of celluloid at half the price of ivory, and are said to be equally elastic, while more durable. Large amounts are used for combs, for the backs of brushes and hand mirrors, and toilet articles; a fine tooth comb made of celluloid is twenty-five per cent cheaper than ivory, while in large pieces, such as the backs of hand glasses, the difference in price is enormous. Among many other articles in which celluloid takes the place of ivory or India-rubber are whip, cane, and umbrella handles, every kind of harness trimmings, foot rules, chessmen, and the handles of knives and forks. Its use in cutlery is said to be especially desirable, as it is not cracked or discolored by hot water.

India-rubber, as a general rule, holds its ground against celluloid, as the latter cannot be sold so cheaply. The celluloid is said to be much more durable, however, and it is superior for pencil cases, jewelry, etc., where gold mountings are used, as it does not tarnish the metal, whereas the sulphur in India-rubber tarnishes gold which is less than eighteen carats fine. The freedom of celluloid from sulphur, and the natural flesh color which can be imparted to it, have caused it to be extensively substituted for India-rubber in the manufacture of dental blanks, or the gums and other attachments of artificial teeth.

Celluloid can be mottled so as to imitate the finest tortoise shell, and its elasticity renders it much less liable to breakage. In this form it is used, like the imitation ivory, for combs, card cases, cigar cases, match boxes, pocket books, napkin rings, jewelry, and all sorts of fancy articles. The substance is employed for similar purposes as a good imitation of malachite and also of amber. It is made into mouth pieces for pipes, cigar holders, and musical instruments, and is used as the material of flutes, flageolets, and drumsticks. For drumheads it is said to be superior to parchment, as it is not affected by moisture in the atmosphere.

As a substitute for porcelain, celluloid is used for the heads of dolls, which can be hammered against a hard floor without danger of fracture. Beautiful jewelry is made of it in imitation of the most elaborately carved coral, reproducing all the shades of the genuine article.

One of the large manufacturing companies is employed exclusively in the making of optical goods, using celluloid in place of tortoise shell, jet, etc., for the frames of spectacles, eye glasses, and opera glasses. The material is extensively used for shoe tips, protecting the toe as well as metal tips, and having the appearance of patent leather. By shoemakers it is also used for insoles. Large quantities of thimbles are made of it, and it is said to be the best material known for emery wheels and knife sharpeners. As a ground for paintings, celluloid has all the advantages of ivory, and photographs can be taken on it which are alleged to be superior to ivorytypes.

Within the last year and a half another branch of celluloid manufacture has been developed which promises to reach enormous proportions. This is the use of celluloid as a substitute for linen or paper in the making of shirt cuffs, collars, etc. It has the appearance of well starched linen, is sufficiently light and flexible, does not wrinkle, is not affected by perspiration, and can be worn for months without injury. It becomes soiled much less readily than linen, and when dirty is quickly cleaned by the application of a little soap and water with a sponge or rag. For travellers and for wear in hot weather this celluloid linen is especially convenient. It has lately been much improved by the introduction of real linen between two thicknesses of celluloid. Shirt fronts have been made of it, as well as cuffs and collars, and it is believed that these will prove equally desirable.

Celluloid has been experimented with as a material for neckties, and although the trials have not yet been very satisfactory, it is thought that they will eventually be successful. For hat bands and hat sweat bands it is a trifle more expensive than the materials commonly used, but it is said to be better, as it does not become rusty or greasy. It has also been used lately for watch-cases.

There is a large export trade in celluloid articles to Cuba and South America, and this is constantly increasing. They are not sent to Europe, as the right to manufacture and sell them there has been sold to a foreign company, which has a factory in France.—*Scientific American.*

---

### ARTICLE VII.

### *Recent Experiments with "Laughing Gas."*

